# Stigma Experienced by Parkinson's Disease Patients: A Descriptive Review of Qualitative Studies

**DOI:** 10.1155/2017/7203259

**Published:** 2017-01-24

**Authors:** Marina Maffoni, Anna Giardini, Antonia Pierobon, Davide Ferrazzoli, Giuseppe Frazzitta

**Affiliations:** ^1^Psychology Unit, Istituti Clinici Scientifici Maugeri, IRCCS Montescano (PV), Pavia, Italy; ^2^Parkinson's Disease and Brain Injury Rehabilitation Department, Moriggia-Pelascini Hospital, Gravedona ed Uniti, Italy

## Abstract

Parkinson's disease (PD) is a neurodegenerative disease characterized by motor and nonmotor symptoms. Both of them imply a negative impact on Health-Related Quality of Life. A significant one is the stigma experienced by the parkinsonian patients and their caregivers. Moreover, stigma may affect everyday life and patient's subjective and relational perception and it may lead to frustration and isolation. Aim of the present work is to qualitatively describe the stigma of PD patients stemming from literature review, in order to catch the subjective experience and the meaning of the stigma construct. Literature review was performed on PubMed database and Google Scholar (keywords: Parkinson Disease, qualitative, stigma, social problem, isolation, discrimination) and was restricted to qualitative data: 14 articles were identified to be suitable to the aim of the present overview. Results are divided into four core constructs: stigma arising from symptoms, stigma linked to relational and communication problems, social stigma arising from sharing perceptions, and caregiver's stigma. The principal relations to these constructs are deeply analyzed and described subjectively through patients' and caregiver's point of view. The qualitative research may allow a better understanding of a subjective symptom such as stigma in parkinsonian patients from an intercultural and a social point of view.

## 1. Introduction

At first blush, Parkinson's disease (PD) is characterized by motor symptoms. Indeed, the four cardinal features of this pathology are identified in the tremor at rest, rigidity, akinesia (or bradykinesia,) and postural instability [1]. Nevertheless, nonmotor symptoms are as much as relevant, even if invisible or not immediately detectable, and they often imply a negative impact on Health-Related Quality of Life (HRQoL) [2, 3]. A significant one is the stigma experienced by the parkinsonian patients and their caregivers. In fact, this phenomenon has not a secondary importance: stigma appears to provide a determinant contribution to HRQoL in patients with PD [4]. Moreover, stigma may characterize everyday life with a gloomy filter, marked by disability and isolation [5].

The general meaning of the word* stigma* is linked to a complex experience concerning a devaluating, discriminant, and discomfort feeling. According to one of the first contemporary conceptualizations of this construct, stigma is an attribute implying a discredit of the subject who is considered “bad, or dangerous, or weak. She/he is thus reduced in our minds from a whole and usual person to a tainted, discounted one” [6, p. 3]. A stigmatized person, that is a person with stigma, is someone who appears changed and different from what is considered normal and accepted. As a consequence, the subject could be isolated and the social identity may be deeply threatened and mined, too [7].

When stigma is present in PD, it originates from the interface of the patient with the outside world [4]. That is, it is not only an individual construct but rather a social one. Indeed, it is a sort of mark underlining the deviant nature of the stigmatized subject from the perspective of those who are the stigmatizers [8]. Indeed, despite different theories, approaches, and models developed over the years [9–11], there is always a social component to consider when speaking about stigma. Therefore, stigma is a complex phenomenon due to the interaction between a context and a subject who receives a devaluating mark [7].

Scales and tools have been developed in order to better describe and quantify stigma in chronic illnesses, such as PD ([12, 13]; for a review, [14]). Nevertheless, being a personal experience, stigma is difficult to be objectively described. The core meaning of this construct could be better defined by collecting the subjective point of view of PD patients [15].

Aim of the present work is to qualitatively describe the stigma of PD patients stemming from literature review, focusing on qualitative international research.

## 2. Method

The authors applied a research strategy to sum up a descriptive overview of the complex and motley experience of stigma in PD linked both to disabling physical conditions and to social, relational, and communicative obstacles. The review was restricted to qualitative published articles in order to catch the subjective experience and meaning of the stigma construct.

Literature review was performed on PubMed database and Google Scholar (keywords: Parkinson Disease, qualitative, stigma, social problem, isolation, discrimination) and was not limited to any country neither to any period of time. 26 papers were identified. The authors read all materials in order to identify where the experience of a devaluating and discriminant feeling linked to the PD effectively emerged and/or was exhaustively discussed. The authors excluded papers in which stigma was an introductive or very marginal, not informative theme. Duplicates were also deleted. Another exclusion criterion adopted was the focus on quantitative reports. Consensus was reached by a vis-à-vis discussion and followed by email discussions. An article was included in the study only when a general consensus was provided.

After that procedure, 6 articles were selected. Subsequently, other 8 additional qualitative studies were selected from references to chosen articles, according to the same methodology previously adopted. At the end of the process, 14 articles were identified to be suitable to the aim of the present overview.

In order to understand and identify the stigma experience, two authors (MM, AG) read the selected 14 articles and took notes of each aspect linked to the construct, discussing online and trying to reach an agreement. In order to reach a consensus among all authors, an iterative process of continuous analysis of data was applied. First, an initial or open coding procedure was carried on, afterwards MM and AG extrapolated the meaningful issues, and AP, DF, and GF read the articles verifying the coherence among the extrapolated key words and reported themes. All emerging issues were considered in the review, considering that even the less quoted experience could contribute to better understanding of the complex stigma phenomenon. All authors contributed to conceptualize stigma experience, critically organizing the emerging themes into major categories. Memos, diagrams, and maps were used as tools enabling data sharing and to reach a consensus.

Results are presented in a descriptive/narrative way, describing the thematic issues linked to stigma experience; in order to simplify comprehension, the results and the identified categories are also presented in a table and graphically summed up.

## 3. Results

As for other chronic and progressive disabling diseases, PD drove patients to experience stigma day after day. From patients' point of view, stigma appears as a complex construct with multiple undesirable facets. This emerged from the plethora of expressions linked to stigma used by patients addressing PD: shame, disgrace, embarrassment, feeling dishonorable, and feeling awkward, terrible, or horrible, and so on [5, 16, 17, 23, 24, 28].

**Figure 1:**
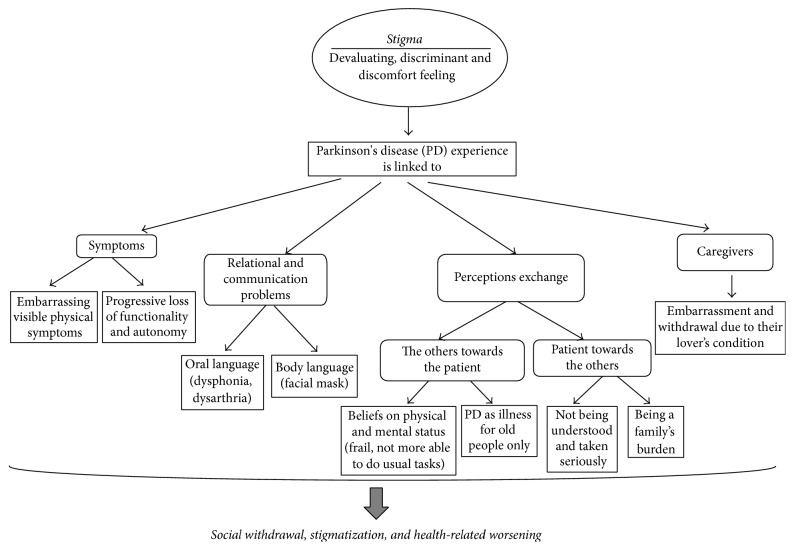
Stigma's core constructs in Parkinson's disease.

In Table 1, the 14 articles included in our descriptive review are described. In order to organize data that emerged from literature review, results were divided into paragraphs according to identified key words; results are quantitatively described in Table 2 and represented as a whole in Figure 1.

### 3.1. Stigma Arising from Symptoms

Elements and conditions determining stigma are different. One of the main causes identified by patients of this phenomenon are motor, physical, and visible symptoms [5, 16, 18–20]. It is not casual that ancient Greeks coined the term stigma referring to “*bodily signs* designed to expose something unusual and bad” [6, p. 1]. Indeed, symptoms are impossible to hide and become a clear and concrete statement of the subjective perception of a capricious disease that speaks by means of the body [17, 21]. In the selected article, the perception of stigma emerges directly linked to PD symptoms and their manifestation in public [18]. In this regard, Israeli women describe their visible body as a traitor, since it unscrupulously reveals PD to the public [16]. That is, body becomes a servant of PD acting by means of its visible symptoms. In this regard, Hermanns's ethnographic approach reveals that the observable traits of disturbances such as drooling, balance difficulties, shaking problems, and other similar symptoms are additional challenges for the patient [5]. PD disrupts the experience of an autonomous and integrated human being due to the exterior signs of the illness condition [22]. Moreover, the deteriorated body image provokes feelings of shame and embarrassment leading to isolation [19, 20].

Nevertheless, stigma is not only linked to the changing exterior image of PD patient but also to the progressive loss of functionality. The contribution of PD to the stigma experience is double: an undesirable self-image and a loss of autonomy and self-efficacy. Indeed, when asking to freely tell their life history with PD thorough in-depth interviews, subjects describe their symptoms as a matter of shame because of the physical dependence and the need for help to do even the simplest tasks [16, 23, 25]. Stigma may arise from the consciousness of the awkwardness and inability to perform not only usual work activities but also simple motor actions [24]. An impoverishment of physical functionality conducts to a reduction of activity and social engagement linked to stigma perception [18].

### 3.2. Stigma Linked to Relational and Communication Problems

Stigma experience arises also from the hindrances to communication and relational life imposed progressively by PD. Indeed, the relationship with the others becomes complex and contradictory since the PD patient has to find the right balance between contact and distance [21].

First of all, relations and communications are a matter of complaint; patients state to be frequently mislabelled, for example, as drunkard [5]. Moreover, the delayed thinking process and the difficulty to convey beliefs easily may cause a subjective experience of frustration and isolation, since the others take their own decisions without waiting for the patient's feedback [26]. Communication changes may have an impact on the patients' and their caregivers' life; subjects attribute to voice and articulation changes many disabling impacts: formulation problems and attention difficulties and subjective feelings of frustration and neglecting linked to withdrawal [27]. Indeed, all these issues conduct the PD patient to remain confined at home, where she/he feels normal and more comfortable since they can bypass the comparison with the outside society [5, 19].

However, communication is not only a matter of speaking. Human beings communicate by means of gesture and body language too. In this regard, face is a sensitive topic to PD patients. Indeed, one of the most visible and undesirable features is the typical rigid and unexpressive face of PD patients, the facial mask. Patients describe other people's difficulty to decipher their mute expression and this condition inexorably causes isolation of the stigmatized person, who perceives a progressive sense of alienation and disconnection from the others [5, 24].

### 3.3. Social Stigma Arising from Perceptions Exchanges

Stigma is a complex matter of feeling and perceptions; the interface between inside and outside is determinant. This means that the contribution of patients and caregivers is equally important and the starting point of a stigmatizing perception is usually shared without clear boundaries.

#### 3.3.1. The Others' Perceptions towards the PD Patient

Taking into account the others' point of view, Hermanns highlights that a PD patient notices to be seen as frail by the others [5]. Moreover, subjects describe a perceived sense of unease and uncomfortable feelings of individuals that are in front of their physical problems and that cannot escape from their sight [24]. Indeed, it is the response of the others that is a matter of shame [23]. When the disease proceeds and disability increases, the patient has to struggle with the experience of marginalization and isolation due to the forced abandonment of work and responsibilities [21].

As to symptoms (i.e., hallucinations), patients may avoid telling their family members, since they worry they could perceive them as cognitively impaired or not being reliable anymore [26]. Indeed, a group of women highlights the difference when speaking of functional aspects that are more socially accepted than of mental and degenerative issues: PD is linked to a diffused and stigmatizing belief of being a disease characterized by a cognitive impairment transforming patient into an insane [16].

Another main reason of social stigmatization is linked to common belief that PD is a disease only for old people; this prejudice may be strong in society, including family members [5, 16]. Interestingly, from a qualitative research conducted within a rural zone of Tanzania, PD is called “old age illness” in Swahili language due to the common prejudice of the age range in which PD may appear [28].

#### 3.3.2. The PD Patient' Perceptions towards the Others

From the patient's perspective, stigma may be linked to patient's metaperceptions of the other people's beliefs towards PD [18]. Patients frequently complain to be misunderstood or even not to be understood and be taken seriously from the outside normal society [5]. A reason of misunderstanding may stem from the fluctuating nature of PD symptoms that leads family members and formal caregivers to believe she/he is pretending [26]. Moreover, some interviewed PD patients complain not to receive the right time to express themselves by the others who replace them in discussions or decisions, without fully understanding their communication difficulties [27].

Stigma arises also from the patient's perception to be a burden for caregivers, also due to the uncertain progression of the disease [5, 16, 18, 24, 26]. Subjects may report feelings of guilt and selfishness towards their caregivers due to the increasingly demanding request of special attention in the daily chores [25]. Moreover, PD patients experience a stigma feeling due to the change or loss of their social roles: they are not the providers of their families and they are often forced to leave their workplace [24].

### 3.4. Caregiver's Stigma

Stigma not only is an experience of the patients but rather may be also a feeling characterizing the family caregivers. PD is something unwanted that marks all the family, leading to difficulties also in public settings and to undesirable feeling of shame and pity [24]. In this regard, Mshana et al. show that, in rural communities of Tanzania, there is a trend to stigmatize the entire PD patient's family because of the dishonorable condition experienced [28]. Indeed, the PD patient's family members may be totally absorbed by taking care of the ill family member, since they are not able any more to take part in social and working life of their community. Equally, also in a European context, the patient's families have been frequently led to a forced withdrawal, in particular during meal time when it becomes difficult to invite guests [25] or at least dealing with the embarrassing and visible symptoms of their ill family member [24].

## 4. Clinical Implications

Being in contact with the patient and discovering her/his experience and inner psychological needs may guide health care professionals and caregivers to take care of the ill person in a more fitted and tailored manner. In fact, by understanding a disease as a whole, from a holistic point of view, one could provide clues to be more effective in patient's management. The capricious and unpredictable nature of this progressive neurological disorder makes the comprehension of a patient's experience and psychosocial correlates even more fundamental. Stereotypes, misunderstandings, shame, isolation, discriminations, and stigmatization are a silent, partly visible, and partly invisible phenomenon, which is necessary to be considered [5]. Indeed, stigma has an important negative effect on the illness progress and management: it may contribute to avoiding or interrupting treatment, as well as to manifesting depressive symptoms [29, 30]. Although dedicated to mental illness and presenting still controversial results, specific psychotherapeutic approaches to stigma seem to be effective, enhancing skills to deal with self-stigma through self-esteem, empowerment, and help-seeking behavior enhancement [31, 32]. Further studies on patients with chronic diseases intended to implement a focused intervention on stigma, may be deserved, adapting protocols and outcome measures on this specific population.

Moreover, stigma has an intrinsic complexity that deserves to be better understood [30]; there is an important need to reach and educate who is foreign to being chronically ill and as an outsider nurtures the stigma phenomenon. By disseminating information and by educating the others, starting from the informal caregivers, we could treat properly this source of sufferance, in line with the WHO ICF model, where disability stems from the interaction of a health condition with personal and environmental factors [33].

Finally, the choice of a holistic and multidisciplinary treatment of all symptoms of PD appears of great importance to guarantee a satisfying health management of the patient [17, 34]. The need to focus on nonmotor symptoms in PD, which stigma belongs to, moves a step forward to a tailored patient-centered medicine, enabling the health professionals to see the patient as a person, living in an everyday life.

## 5. Conclusions

The social consideration and attitude towards a disease are important, since they contribute in determining the environment in which the patient has to live and interact in [5, 35]. Indeed, disability stems from the interaction between the individual and the environment [36]. Stigma is a complex phenomenon well attested and in need of comprehension in the context of chronic diseases and PD is not an exception [37]. Even if stigma could be a silent and invisible phenomenon [5], it may have direct relevant impact on HRQoL [4].

Our focus on qualitative approaches could contribute to sustain a subjective insight into patient's experience [15]. In fact, patients are the most trustworthy witnesses of their lives. They are the main protagonists of their changing illness experience: day after day, they live on with their body and continuously come to terms with the PD [5, 17].

Stemming from our review stigma could be considered as a nonmotor symptom as relevant as the other ones. In fact, stigma is not only a feeling of shame and embarrassment arising from a self-perception of inadequacy due to loss of autonomy and visible symptoms but also an experience related to the attitudes and beliefs of the social context towards the PD patient who is stigmatized and forced to withdrawal. That is, it is the negative or positive response of the outside world that may do the difference. Indeed, according to the recent ICF conceptualization, disability is not only a state linked to personal limitations and impairments but also a condition interconnected with the environment and the interface with it [36, 38].

To date, what PD patients and their caregivers seem to experience is a mark [8], a shameful sign of different needs and impaired behaviors. Indeed, PD manifestations break social rules and all what is normally attended by a healthy social community [23]. Further qualitative studies on this topic are needed in order to better understand a subjective symptom as stigma in parkinsonian patients from an intercultural and a social point of view.

## Figures and Tables

**Table 1:** Study characteristics of the 14 articles included in the qualitative review.

Study	Location of patients' recruitment	Number of participants	Qualitative methods	Study aim
Nijhof, 1995	Amsterdam, The Netherlands	23 PD pts (10 F; 13 M)	In-depth interviews with qualitative analysis of content	To explore PD subjective interpretations
Posen et al., 2000	Tel Aviv, Israel	15 PD pts (F)	Sessions of psychoeducational work-group (MacKenzie and Livesley, 1983)	To describe the PD experience in a female work-group
Sunvisson and Ekman, 2001	Sweden	11 PD pts (no gender details)	Interviews during a period of 2 years and phenomenological data analysis	To elucidate environmental influences on lived PD experiences
Van Der Bruggen and Widdershoven, 2004	/	4 novels	Existential-phenomenological analysis of narrative materials of PD patients	To catch the meaning of being a PD patient
Bramley and Eatough, 2005	Nottingham, UK	1 PD pts (F) (single case study)	Semi-structured interviews analyzed using interpretative phenomenological analysis (IPA)	To catch the subjective PD daily experience
Miller et al., 2006 (a)	Sunderland, UK	37 PD pts (14 F; 23 M)	In-depth interviews with qualitative analysis of content	To study changes in communication impact on daily PD patients' lives
Miller et al., 2006 (b)	Sunderland, UK	37 PD pts (14 F; 23 M)	In-depth interviews with qualitative analysis of content	To establish if and how changes in swallowing impact on daily PD patients' lives
Mshana et al., 2011	Mwanza, Tanzania	28 PD pts, 28 caregivers, 4 health workers, 2 traditional healers (no gender details)	In-depth interviews and focus groups	To detect how PD is perceived and treated in a rural African population
Chiong-Rivero et al., 2011	USA	48 PD pts (26 F; 22 M) 15 caregivers (13 F; 2 M)	Focus groups and one-on-one interviews	To collect Health-Related Quality of Life consequences of Parkinson's disease from the patient's and caregivers' perspective
Hermanns, 2013	Texas, USA	14 PD pts (7 F; 7 M)	Ethnographic approach using interview data, participant observations, and fieldwork (2-year exposure)	To discuss the visible and invisible stigma
Soleimani et al., 2014	Iran	10 PD pts (3 F; 7 M)	Semistructured, face-to-face interviews and content analysis approach	To explore the effects of PD on people's social interactions
Soundy et al., 2014	/	37 qualitative articles (review)	Metaethnography	To summarize and to synthesize qualitative studies concerning the PD experience and perception
Giardini et al., 2016	Montescano (PV), Italy	27 PD pts (14 F; 13 M)	Semi-structured interviews with PD patients analyzed using the Grounded Theory methodology	To qualitatively describe the rehabilitation experience of PD inpatients
Soleimani et al., 2016	Iran	17 PD pts (7 F; 10 M)	Semistructured, face-to-face interviews and content analysis approach	To explore the primary concerns and perceptions of daily PD patients' lives

Legend: PD = Parkinson's disease; Pts = patients; F = female; M = male.

**Table 2:** Thematic issues related to stigma experience identified in the reviewed articles.

Thematic issues	Reference number of each reviewed article
*Symptoms*	[5, 16–22] [5, 16, 18, 23–25]
Embarrassing visible physical symptoms	
Progressive loss of functionality and autonomy	

*Relational and communication problems*	[5, 19, 26, 27] [5, 24]
Oral language (dysphonia, dysarthria)	
Body language (facial mask)	

*Perceptions exchange*	
The others towards the patient	[5, 16, 21, 23, 24, 26] [5, 16, 28]
Beliefs on physical and mental status	
PD as an illness for old people only	
Patient towards the others	[5, 26, 27], [5, 16, 18, 24–26]
Not being understood and taken seriously	
Being a family burden	

*Caregivers*	[24, 25, 28]
Embarrassment and withdrawal due to their lover's condition	
